# Sensory basis of speech motor learning and memory

**DOI:** 10.1073/pnas.2525468123

**Published:** 2026-04-24

**Authors:** Nishant Rao, Rosalie Gendron, Timothy F. Manning, David J. Ostry

**Affiliations:** ^a^Yale Child Study Center, Yale School of Medicine, New Haven, CT 06511; ^b^Department of Psychology, McGill University, Montreal, QC H3A 1G1, Canada

**Keywords:** human speech production, auditory cortex, somatosensory cortex, motor cortex, transcranial magnetic stimulation

## Abstract

Changes to speech offer a quantifiable means to assess speech motor learning, and the resulting memory is thought to be motor in nature. Here, we evaluate this idea and show instead that memory for speech movements has a sensory basis. Speech motor learning, using altered auditory feedback, provides an experimental model to address this question as it involves auditory, somatosensory, and motor components to learning. Transcranial magnetic stimulation was used to disrupt auditory (superior temporal gyrus, STG), posterior somatosensory (S1), or motor (M1) cortex following speech motor learning. Retention tests were conducted 24 h later. It was found that following disruption of either STG or S1, motor memory retention was impaired whereas disruption of M1 led to retention that was no different than that of a no TMS control condition. The effects of disruption were specific to speech motor learning and did not interfere with speech production per se. Taken together, the findings support the notion that plasticity in the sensory cortex, both auditory and somatosensory, is necessary for speech motor learning and memory. In speech, changes to sensory systems enable the production of newly learned movements.

## Significance

The present study tests the causal contribution of cortical brain regions to speech motor memory. Using magnetic brain stimulation, it is shown that disruption of the sensory cortex, either auditory or somatosensory, impaired speech motor memory retention. In contrast, disruption of the primary motor cortex did not produce an impairment. These findings show that the changes to sensory cortex are necessary for speech motor learning and memory.

Speech motor learning involves a network of frontal, parietal, and temporal cortical brain regions (1–3). Identifying which among these areas are key to speech motor learning and motor memory retention is challenging. Some areas could be causally involved in the process whereas others might be activated by virtue of their connections with key areas. Activity in the primary motor cortex (M1), of necessity, changes during learning to enable the production of new movements. However, on its own, this is not evidence that the motor cortex causally contributes to learning as plasticity elsewhere might drive the new movement and new motor cortical activity through projections to the motor cortex. Here, we investigate both sensory and motor cortical contributions to speech motor memory by individually disrupting auditory, somatosensory, and motor cortical activity.

Studies aimed at determining regions that causally contribute to learning, and to speaking and listening more generally, have used brain stimulation which has often been applied to M1 prior to or during motor learning (4–11). A problem in interpreting any subsequent impairment in learning following stimulation over M1 is that stimulation of the motor cortex can produce changes to perception, movement, and cortical excitability, thereby impairing learning indirectly as a consequence (7, 9, 11). A cleaner test for the participation of an area in motor learning and memory involves an examination of the effects of stimulation following learning on subsequent retention. By disrupting activity immediately following learning, one targets the products of learning, that is, any learning-related changes which may have occurred. If an area is involved in this process, interference with the products of learning should result in an impairment in retention. Stimulation following learning also rules out the possibility that stimulation has produced an impairment by disrupting the capacity to learn.

The present study applies continuous theta-burst magnetic brain stimulation (cTBS) following speech motor learning to infer causal involvement of sensory and motor brain areas in this process. Adaptation to altered auditory feedback, which involves compensation for real-time vowel frequency shifts, serves as an experimental model for learning. We have previously described the behavioral underpinnings of speech motor learning and retention using this technique (12). In the current study, stimulation was delivered following learning, either to the left hemisphere tongue area motor cortex (M1), to the left somatosensory cortex (S1) overlying the postcentral sulcus, or to the left auditory belt area of the superior temporal gyrus (STG) in a region identified by previous studies to encode speech oral sounds (13–15). Tests for retention were conducted 24 h later. We find that disruption of either auditory or somatosensory cortex results in an impairment in retention, whereas disruption of the motor cortex does not. This indicates that speech motor learning and memory are substantially sensory in nature.

## Methods

### Participants.

Data from 71 healthy young adults are reported in this work. Of these, data from 15 control participants were taken from our previously published manuscript (12). All participants were screened for contraindications to TMS, and none reported any speech or hearing disorders. Participants also provided written informed consent, and the study procedures were approved by the Institutional Review Boards of McGill University and Yale University. Data from eleven of these participants were excluded due to an absence of statistically reliable learning on visit 1 before TMS. Collectively, data from 60 participants (21 males; age mean ± SE: 23.25 ± 0.49) were considered for subsequent analyses.

### Experimental Setup.

**Fig. 1.**
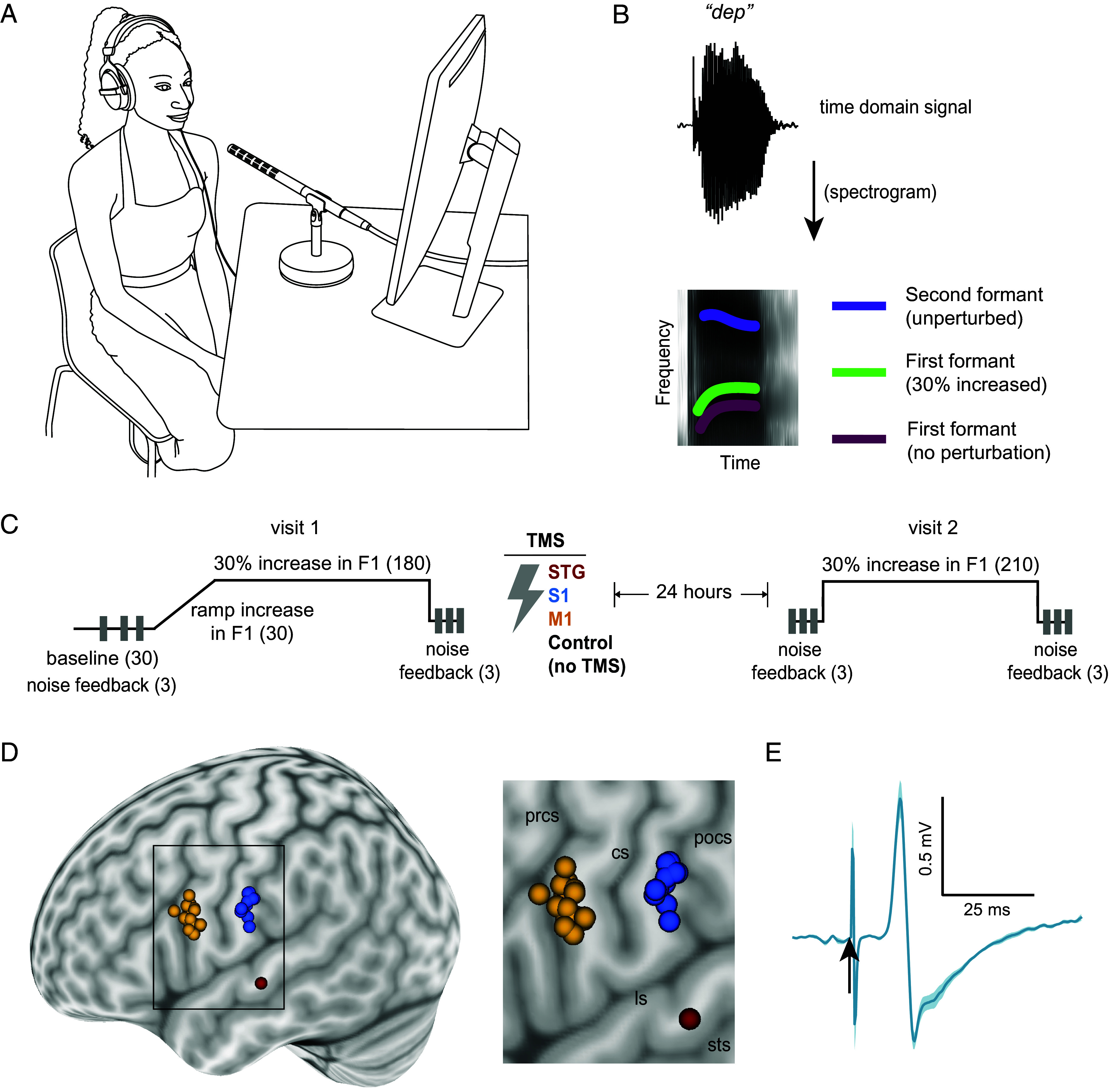
Experimental setup. (*A*) Participant in the speech motor learning task. (*B*) For an example word stimulus “dep,” the *Upper* plot shows the time-domain signal; the *Lower* plot shows the spectrogram of the same signal, with the traces showing schematic of first and second formant frequencies. (*C*) Experimental design in which rectangular gray patches indicate noise feedback trials, and the numbers in brackets indicate number of trials in that block. (*D*, *Left*) TMS stimulation sites for M1, S1, and STG conditions; (*Right*) zoomed view of the rectangular area shown at the left (cs: central sulcus, prcs: precentral sulcus, pocs: postcentral sulcus, ls: lateral sulcus, sts: superior temporal sulcus). (*E*) Motor evoked potential (right genioglossus muscle) from left hemisphere tongue area motor cortex. Solid line and shaded region indicate mean and SE respectively, the vertical arrow indicates the TMS single stimulation pulse. Panels *A* and *B* adapted from ref. 12, which is licensed under CC BY 4.0.

The experimental setup to assess speech motor learning and memory was identical to that described in a previous study (12). Participants were seated and read words presented on a display monitor, as shown in Fig. 1*A*. Their speech was recorded using a unidirectional microphone (Sennheiser). Participants were presented with their speech acoustical signal as auditory feedback in real-time through headphones (Beyerdynamic DT770M), either with no manipulation or with frequency shifted auditory feedback using Audapter (16) integrated with MATLAB. An audio mixer (MOTU/TASCAM) enabled separate control of gain to adjust the microphone and headphone signals.

The study also involved both single pulse and continuous theta burst transcranial magnetic stimulation (cTBS). Single pulse TMS was delivered using a Magstim *200^2^* stimulator, and cTBS using a Magstim *Rapid^2^* TMS unit (Magstim, Whitland, United Kingdom). TMS coil motion was tracked using the Brainsight Neuronavigation system (Rogue Research, Montreal, Quebec, Canada).

### Speech Motor Learning and Retention Task.

The experimental auditory manipulation employed in this study is indicated in Fig. 1*B*. The task manipulated the vowel structure of speech to elicit learning and retention. Vowels, in acoustical terms, comprise vocal tract resonances known as formants, where the first and second formants (F1 and F2 respectively) account for the most acoustical energy. The primary experimental manipulation involved the alteration of F1 while not perturbing F2 and providing the altered speech as auditory feedback to the participants in real-time via headphones. This paradigm is known to elicit a learning process during which participants compensate for an experimentally increased F1 by reducing F1 in their vocal output values over multiple trials. To limit the linguistic influence on the speech motor learning and memory, we used pseudowords (“bep,” “gep,” and “dep”) as stimuli throughout. We have previously established that learning and retention using these stimuli are robust to abrupt or gradual introduction of perturbation and similar when assessed either 8- or 24-h following learning (12). The sequence of these stimuli was pseudorandomized for each participant.

A schematic of the experimental design is shown in Fig. 1*C*. Each participant came for two visits separated by ~24 h. During visit 1, they first underwent a block of practice trials to familiarize themselves with the experimental setup, followed by a block of 30 baseline trials with no perturbation to the formant frequencies. Interspersed within the baseline block were three additional trials where participants received speech-masked noise feedback instead of their speech feedback (referred to as noise feedback trials). Restricting participants’ speech auditory feedback helps to estimate the feedforward component of speech production while masking the noise feedback with a speech envelope helps reduce the Lombard effect (tendency to be louder when hearing noise). Following the baseline and noise feedback trials, participants went through 30 trials in a gradual ramp phase in which F1 frequencies in their auditory feedback were increased on a trial-by-trial basis until they reached a 30% change. Following this phase, participants received 180 trials during which their auditory feedback was maintained at 30% increased F1. Visit 1 concluded with three speech-masked noise feedback trials to assess the extent of learning in absence of speech error feedback. Visit 2 started with three noise feedback trials, followed by 210 trials with 30% increased F1 throughout with, as in the first session, no change in F2, and ended with three noise feedback trials. The amplitude of the auditory feedback signal was increased for all participants so they could not hear their own voice beyond that fed back through the headphones. The participants also received instructions to speak softly and consistently and stay ~15 cm from the microphone.

### Transcranial Magnetic Stimulation.

Participants were seated at rest during the TMS procedure. In the motor and somatosensory cortex conditions, we began by identifying tongue area motor cortex. First, the participant’s tongue was cleaned and dried using sterile oral sponges. Two 12 mm square Ag/AgCl disposable hydrogel electrodes surfaces were placed on the right side of the tongue, contralateral to the site of stimulation. The electrodes were placed above and below the tongue at about 0.5 cm lateral to the tongue’s midline to capture activity in the genioglossus muscle. The location of the hotspot was determined as the position over the lateral precentral gyrus at which stimulation elicited the largest repeatable genioglossus MEPs. This was done using single-pulse TMS with the participant’s tongue at rest. Fig. 1*E* illustrates MEPs obtained from a representative participant when stimulating the M1 hotspot. If MEPs could not be found at rest, participants were asked to push the tongue gently against the teeth to engage tongue muscles. MEPs were found in all participants.

The causal involvement of candidate cortical regions in speech motor learning and memory was assessed by assigning participants to either M1, S1, STG cTBS conditions or to a no TMS control condition. cTBS was applied at the end of visit 1, either to the M1 tongue area hotspot or to a point ~2 cm posterior over the posterior postcentral gyrus (referred to as S1). The M1 and S1 procedure used here was similar to that in our previous work and in accordance with previously reported face motor and sensory locations on the pre- and postcentral gyrus (15, 17–19). As an auditory stimulation target, we first determined MNI coordinates of a previously identified region in the auditory belt area of the superior temporal gyrus which was responsive to vowel characteristics of speech sounds (13, 14, 20). Next, each participant’s head was coregistered with the MNI brain template using Brainsight (Rogue Research, Montreal, Canada), and stimulation was delivered to the MNI coordinates localized in this manner. The mean MNI coordinates (x, y, z format) were (−80.08, 8.12, 32.43) for M1, (−80.75, −13.91, 35.02) for S1, and (−65.13, −17.25, −0.51) for STG locations (Fig. 1*D*). Participants received two rounds of cTBS, with each round involving bursts of three pulses at 30 Hz, repeated at 6 Hz for a total of 600 pulses at 40% of maximum stimulator output intensity. The intensity was lowered up to 35% if participants expressed discomfort at 40% intensity. Each round was separated by 10-min duration. cTBS delivery concluded the visit 1 and there was no TMS procedure on visit 2.

### Data Analysis.

The speech signal was sampled at 48 kHz and downsampled to 16 kHz. The Montreal forced aligner (21) was used to estimate the vowel boundaries and manually checked when needed. A Burg algorithm using the Praat software (22, 23) was used to extract the F1 and F2 formant frequencies from the speech data. A segment of 40 ms centered at the vowel midpoint was averaged to obtain F1 and F2 values for each trial. The speech utterance was intentionally brief (and not in the form of an elongated utterance), and we have shown previously that the vowel durations in such a paradigm average to ~155 ms, leading to vowel midpoints around ~77 ms (12). Extracting formant values around this timepoint primarily highlights the feedforward component of the speech movements and limits involvement of feedback-based corrective responses within a trial (12, 24, 25).

We verified that vowel parameters of interest were not different across TMS conditions for the baseline phase using ANOVA (α = 0.05). Subsequently, baseline values for each participant were averaged and the percentage change in F1 from baseline was computed to assess the learning and retention separately for each condition. Asymptotic performance for learning and also for relearning was assessed by taking an average F1 change from baseline over the last 30 trials of visit 1 and visit 2 respectively. Retention was assessed as an average of three trials following the first speech feedback trial on visit 2. Speech production per se was quantified by assessing the percentage F1 change from baseline in the noise feedback trials during the baseline block of visit 1, and at the start of visit 2 prior to the retention tests. Tests for differences in experimental conditions were conducted using ANOVA (α = 0.05). Post hoc comparisons were performed using one- or two-sample t-tests with corrections where applicable.

## Results

The primary goal of the study was to investigate the causal involvement of sensory and motor cortical regions in speech motor learning and motor memory retention. Separate conditions involved participants receiving noninvasive disruption of neural activity via TMS over STG, M1, S1, or control (no disruption via TMS) at the end of a speech motor learning task. Their speech motor memory retention was assessed 24 h later to test for the causal involvement of the different cortical regions in this process. There were no differences in baseline speech performance across conditions for either the first formant F1 frequency (F_(3, 56)_ = 1.930, *P* = 0.135, partial η^2^ = 0.094), or for the second formant F2 frequency (F_(3, 56)_ = 1.429, *P* = 0.244, partial η^2^ = 0.071).

### Learning Related Changes to Sensory Cortex Are Necessary for Speech Motor Learning and Memory Retention.

**Fig. 2.**
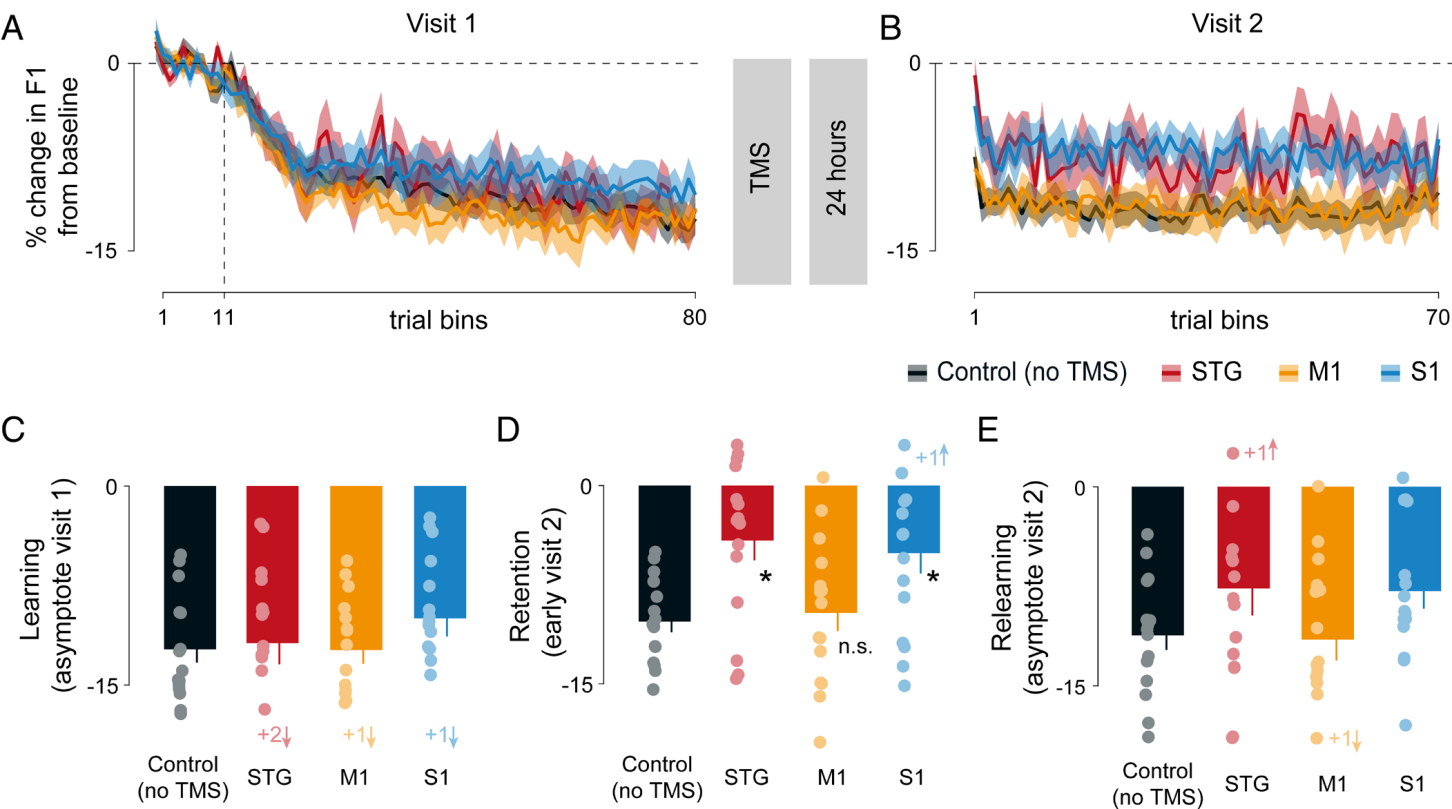
Disruption of the sensory cortex impairs retention of learning. (*A*) Percentage change in F1 from baseline during visit 1 and (*B*) during visit 2. F1 was perturbed in a positive direction, so a change in a negative direction indicates learning. Data are binned and averaged over three consecutive trials without overlap. The vertical dashed line indicates first bin following F1 perturbation, bins preceding that line are baseline utterances with no F1 perturbation. Solid lines and shaded regions indicate mean and SE across participants. (*C*) Percentage change in F1 during visit 1 asymptote (average over last 10 bins defined as learning asymptote). (*D*) Percentage change in F1 during early visit 2 (average over the first three speech feedback trials defined as retention). (*E*) Percentage change in F1 during visit 2 asymptote (average over last 10 bins defined as relearning). Solid circles indicate individual participants’ data, and error bars indicate SE. Asterisk and “n.s.” indicate statistically significant and not significant difference from controls (no TMS) respectively. Numbers followed by a + sign indicate, for visualization purposes, number of data points out of range in the direction shown by **↑** or **↓** arrows.

**Fig. 3.**
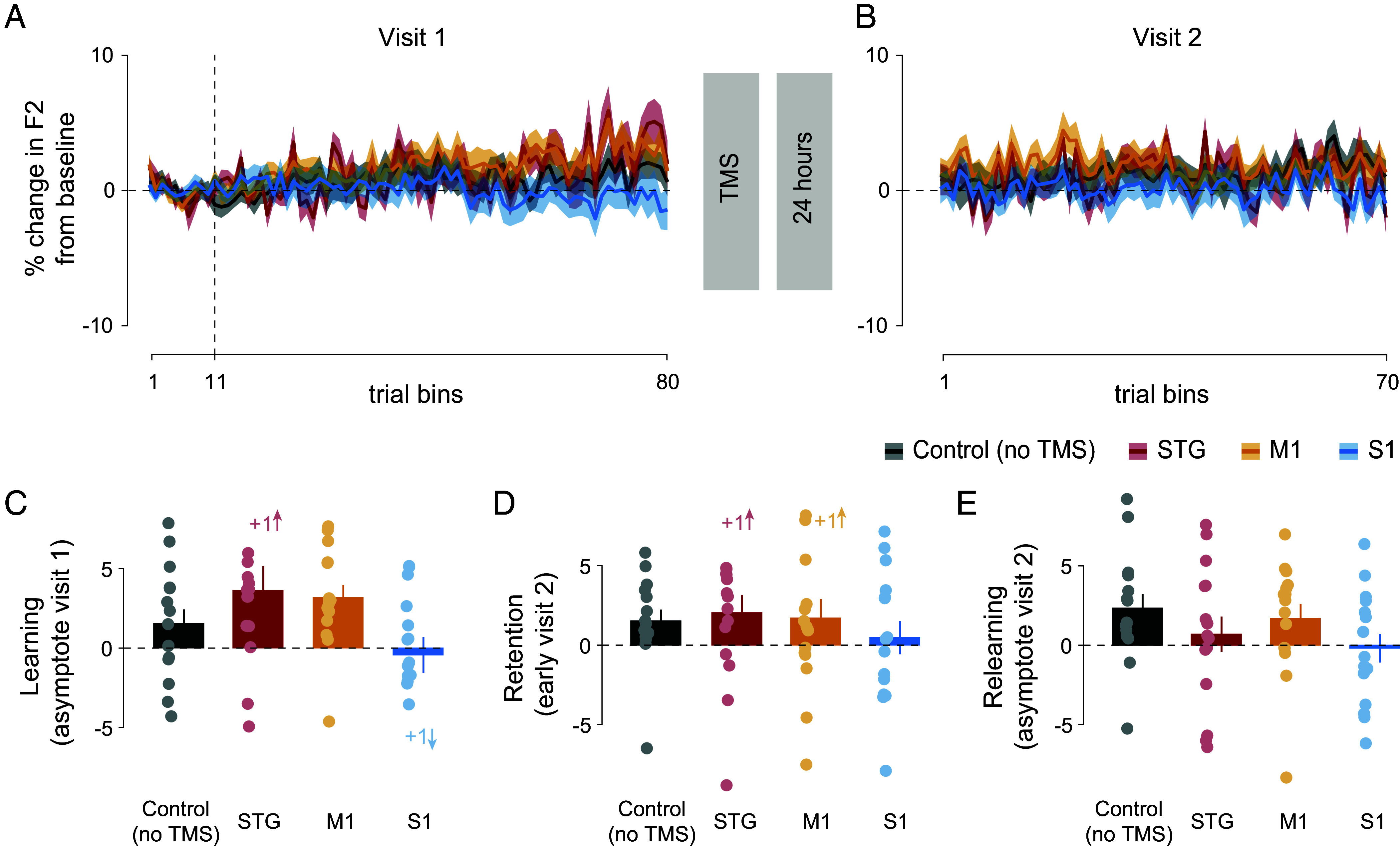
Little effect of TMS on the unperturbed F2 formant frequency. (*A*) Percentage change in F2 from baseline during visit 1 and (*B*) during visit 2. F2 was not perturbed, so values aligned with 0 indicate no change. Data are binned and averaged over three consecutive trials without overlap. Vertical dashed lines indicate the first bin following F1 perturbation, bins preceding that line are baseline utterances with no F1 perturbation. Solid lines and shaded regions indicate mean and SE. (*C*) Percentage change in F2 during visit 1 asymptote (average over last 10 bins defined as learning asymptote). (*D*) Percentage change in F2 during early visit 2 (average over the first three speech feedback trials defined as retention). (*E*) Percentage change in F2 during visit 2 asymptote (average over last 10 bins defined as relearning). Solid circles indicate individual participants’ data, and error bars indicate SE. Numbers followed by a + sign indicate number of data points out of range in the direction shown by succeeding **↑** or **↓** arrows.

The primary results are shown in Figs. 2 and 3. Fig. 2 *A* and *B* show visit 1 (baseline, learning data) and visit 2 (retention and relearning) data respectively. Solid lines indicate percentage change in first formant (F1) from baseline, binned, and averaged over three consecutive utterances without overlap. Colors indicate TMS delivery over STG (red), M1 (saffron), S1 (blue), or control (no TMS; black). The vertical dashed line in the visit 1 plot indicates the start of the learning trials (with auditory perturbation). Shaded regions show the SE across participants for each condition. It can be seen that there are few differences across TMS conditions during visit 1 prior to TMS delivery, as one would expect. However, following ~24 h, speech performance during visit 2 is impaired among participants who received TMS over STG or S1, versus those who received TMS over M1 or no TMS at all (control condition).

Fig. 2*C* shows percentage change in F1 from baseline during asymptotic trials on visit 1 (referred to as learning). Solid circles indicate individual participant’s speech performance, and the error bars show SE across participants. There was no difference in the extent of learning at the end of visit 1 among the four experimental TMS conditions (F_(3, 56)_ = 0.724, *P* = 0.542, partial η^2^ = 0.037).

Fig. 2*D* shows the first three trials of visit 2 (referred to as retention), in a similar color code and axes as in learning. A reliable difference in retention with TMS conditions was observed (main effect of TMS conditions on retention: F_(3, 56)_ = 5.006, *P* = 0.004, partial η^2^ = 0.211). Retention was reduced following TMS over STG versus no TMS (independent t_(28)_ = 3.474, p_corrected_ = 0.006, Cohen’s d = 1.269). Retention in speech performance was also reduced following TMS over S1 versus no TMS (independent t_(28)_ = 2.861, p_corrected_ = 0.012, Cohen’s d = 1.045). Speech motor memory retention following TMS over M1 was similar to that observed in the no TMS condition (independent t_(28)_ = 0.398, p_corrected_ = 0.694, Cohen’s d = 0.145).

Fig. 2*E* shows extent of relearning at the asymptote of visit 2. Although persistent differences appear present over the course of relearning (Fig. 2*B*), a statistical analysis indicated that all TMS conditions converged to a similar level of relearning (no main effect of TMS conditions on relearning: F_(3, 56)_ = 1.680, *P* = 0.182, partial η^2^ = 0.083). Taken together, findings for F1 show similar baseline and learning performance on visit 1 across all conditions and selective impairment of retention compared to controls when TMS was delivered to STG or S1, but not M1.

### No Effect of TMS-Based Disruption on the Unperturbed Formant Frequency.

In previous work, it was observed that there were learning-related changes in the perturbed formant (F1), while changes to the unperturbed formant (F2) were minimal (12). To investigate whether the present TMS-based disruption had any effect on F2, we assessed F2 changes at same instances during the task as when F1 was assessed, that is, end of learning, retention, and end of relearning. Fig. 3 *A* and *B* show percentage change in F2 from baseline, binned, and averaged over three consecutive trials without overlap for visits-1 and 2. Colors differentiate TMS conditions in the same way as for F1 data. It can be seen that none of the TMS conditions show reliable differences at any point over the course of visits-1 and 2.

Fig. 3*C* shows percentage change in F2 from baseline at the end of visit 1 (similar to the learning phase for F1). Solid circles show individual participants’ data and error bars show SE. No reliable differences were observed in F2 percentage change from baseline at the end of visit 1 (F_(3, 56)_ = 2.543, *P* = 0.065, partial η^2^ = 0.120). Fig. 3*D* shows percentage change in F2 from baseline during early visit 2 (similar to retention phase of F1). Here again, no reliable differences were observed during early visit 2 performance (F_(3, 56)_ = 0.404, *P* = 0.751, partial η^2^ = 0.021). Fig. 3*E* shows a similar F2 measure at the end of visit 2 (analogous to the relearning asymptote for F1). It can be seen that percentage change in F2 from baseline failed to show any consistent changes at the end of visit 2 (F_(3, 56)_ = 1.266, *P* = 0.295, partial η^2^ = 0.064). Taken together, these findings indicate no effect of TMS on F2.

### Speech Production Per Se Was Unaffected by TMS.

**Fig. 4.**
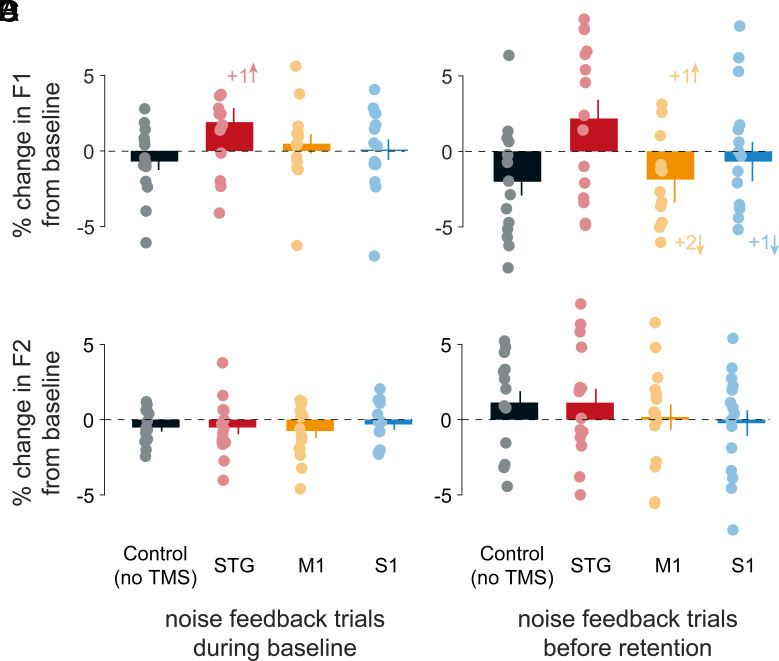
Speech production per se is unaffected by TMS. (*A*) Percentage change in F1 during noise feedback trials at baseline from speech feedback baseline. (*B*) Percentage change in F1 during noise feedback trials in the retention phase from speech feedback baseline. (*C*) Percentage change in F2 during noise feedback trials at baseline from speech feedback baseline. (*D*) Percentage change in F2 during noise feedback trials in the retention phase from speech feedback baseline. Solid circles indicate individual participants’ data, and error bars indicate SE. Numbers followed by a + sign indicate number of individual data points out of range in the direction shown by succeeding **↑** or **↓** arrows.

We assessed whether there was an effect of TMS on speech production as separate from learning by introducing the speech-masked noise feedback trials during various phases of the task. Because our TMS effect was specifically observed during the retention phase of the task in F1, we show speech performance in terms of percentage change in F1 during noise feedback trials (baseline) from speech feedback baseline (Fig. 4*A*), and percentage change in F1 during noise feedback trials (retention) from speech feedback baseline (Fig. 4*B*). Similarly, we show percentage change in F2 during noise feedback trials (baseline) from speech feedback baseline (Fig. 4*C*), and percentage change in F2 during noise feedback trials (retention) from speech feedback baseline (Fig. 4*D*). In earlier work, it has been shown that in the absence of TMS, F1 measures during noise feedback trials at the beginning of the retention phase are no different than those at baseline before learning (12). Here, there were no reliable differences observed in F1 measured during noise feedback trials in the baseline phase (Fig. 4*A*; F_(3, 56)_ = 2.142, *P* = 0.105, partial η^2^ = 0.103) nor during the retention phase (Fig. 4*B*; F_(3, 56)_ = 2.174, *P* = 0.101, partial η^2^ = 0.104). When assessing the same measure for F2, we observed no differences over TMS conditions during the baseline phase (Fig. 4*C*; F_(3, 56)_ = 0.197, *P* = 0.898, partial η^2^ = 0.010), nor during the retention phase (Fig. 4*D*; F_(3, 56)_ = 0.616, *P* = 0.607, partial η^2^ = 0.032). Taken together, speech performance during the noise feedback trials indicates an absence of TMS-based effects on speech production itself, suggesting a disruption of higher-order learning and memory specific mechanisms affected by TMS.

We also assessed F1 and F2 measures during noise feedback trials at the end of learning and relearning phases. As in the baseline and retention phases, no reliable differences in F1 were observed across TMS conditions at the end of visit 1 learning (no effect of TMS conditions: F_(3, 56)_ = 1.783, *P* = 0.161, partial η^2^ = 0.087), nor at the end of the visit 2 relearning phase (no effect of TMS conditions: F_(3, 56)_ = 2.017, *P* = 0.122, partial η^2^ = 0.098). Similarly, no reliable effect of TMS conditions was observed in F2 measures during noise feedback trials at the end of learning (F_(3, 56)_ = 1.490, *P* = 0.227, partial η^2^ = 0.074), nor at the end of relearning (F_(3, 56)_ = 1.837, *P* = 0.151, partial η^2^ = 0.090). It was additionally observed that F1 performance during the noise feedback trials relative to speech feedback baseline was reliably different than zero at the end of learning and relearning (for end of learning: all |t| > 4.300, all p_corrected_ < 0.0007 for each of the four experimental conditions; for end of relearning: all |t| > 3.300 and all p_corrected_ < 0.0050 for each of the four experimental conditions). In contrast, F2 values during noise feedback trials relative to baseline at the end of learning and relearning were not reliably different than zero (all p_corrected_ > 0.05). Taken together, these findings show that the noise feedback trials can quantify the effects of altered speech production when they are present. This underscores the findings of the previous paragraph, that TMS had no effect on speech production per se.

## Discussion

The principal finding is that the superior temporal gyrus (an auditory belt region), and the posterior somatosensory cortex each contribute causally to speech motor learning and retention. On the other hand, the disruption of the primary motor cortex led to speech motor memory that was no different than that observed in a no TMS control condition (Fig. 2). This shows that the TMS effects are regionally specific, and, in the case of the motor cortex, it shows that retention is equivalent to that observed when there is no stimulation at all. F2 was neither affected by F1 perturbation, nor with TMS-based disruption (Fig. 3). Moreover, there was no difference in speech performance between noise feedback trials at baseline or immediately prior to the retention assessment, which indicates that the TMS-based impairment was not due to a failure of speech production on its own (Fig. 4). Taken together, the current study reveals a sensory basis to speech motor learning and memory.

### Sensory Cortical Participation in Motor Memory Retention.

Speech motor learning is known to be associated with activity in a network of brain areas involving left premotor, inferior frontal, and intraparietal areas (1, 2). Speech motor adaptation is also associated with strengthening of connectivity between auditory and somatosensory cortices and other nonmotor areas (3). Notably, these observations have been based either on cortical activity during learning or on resting state functional connectivity, both of which convey correlational changes in brain activity, leaving questions pertaining to causality unaddressed. Our findings of impaired speech motor retention following disruption to sensory cortex (STG and S1), but not primary motor cortex (M1), establishes causality in the context of speech motor learning and memory retention. Note that similar levels of retention following TMS over M1 and a no TMS control condition argue that changes in M1 are not necessary for speech motor memory.

Human motor learning and memory, as assessed by limb movement models, have shown impaired retention following disruption of the somatosensory cortex but not following that of the motor cortex (17, 18, 26). In this context, the finding that disruption of S1, but not M1, leads to an impairment in retention is consistent with that in studies of limb movement. An additional contribution of the current study is the finding that learning related cortical changes are not restricted to somatosensory cortex alone but are also observed following disruption of the STG (housing the auditory belt region), thus providing support for the idea that change in the sensory cortex is a general property of motor learning and memory.

These findings also establish the causal involvement of STG in motor learning and memory, beyond its previously demonstrated sensitivity to a variety of acoustic-phonetic features in addition to vowels, including fricatives, plosives, and nasal speech sounds (13, 14). The site of STG stimulation in the present study was within the auditory belt and parabelt regions (Brodmann Area BA 42 and BA 22) which are known to share connections with the premotor cortex via the arcuate and superior longitudinal fasciculi forming a dorsal pathway (27–29). Connections between speech motor areas and both auditory and somatosensory regions have also been seen using functional connectivity analyses (30). These structural and functional connectivity patterns may be implicated in the transformation of sensory target information into appropriate commands to generate movement (27, 30–35). Our speech motor learning task required the production of compensatory movements for the experimentally perturbed auditory feedback. Therefore, TMS over STG may have disrupted learning-related changes to auditory targets, thereby impairing the retention of the newly acquired speech motor memory.

An important characteristic of learning-related changes to auditory targets is that they are context-specific. In a previous report (12), we found that noise feedback trials, several hours after learning, showed little evidence of retention, whereas substantial retention was observed when speech feedback was available. On the other hand, speech motor memory was successfully retrieved during noise feedback trials when included immediately after learning. This suggests that the newly acquired memory becomes contextually dependent on speech feedback for retrieval with the passage of time between learning and retention tests. Our previous work showed this contextual dependency when retention testing was conducted both 8- and 24-h after initial learning. The cortical bases of this contextualization process remain to be known.

The present findings also indicate causal involvement of the posterior somatosensory cortex in speech motor learning and motor memory retention. The postcentral cortex shares extensive connections with frontal motor areas making it an important node maintaining learning-related changes needed to generate compensatory movements (36–39). In other work using fMRI, speech motor learning has been shown to be directly associated with increased functional connectivity in the areas integrating somatosensory and auditory information (40). It remains to be determined whether disruption of STG or S1 in the present study led to an impairment by introducing a break in an interconnected sensory control circuit or because these areas act separately on frontal motor areas.

Besides memory retention, the speech motor adaptation task employed in the current study also involved both: single word reading and sensory error processing. STG and posterior postcentral gyrus, along with additional areas, were found in other work to be active both during speech production and speech error correction (41, 42). Single word reading, as used here, results in activity in the primary motor cortex, including both medial and lateral premotor areas, auditory cortex, including the auditory belt region and ventral somatosensory cortex, as well as anterior insula and the cingulate motor area. Subcortical activity is also observed in basal ganglia input and output zones (putamen and globus pallidus) and cerebellar cortex lobules VI and VIII.

Sensory error processing, as would be needed for speech motor adaptation, has been studied using cortical recordings and behavioral responses in stroke patients (43, 44). Responses to auditory pitch perturbations in speech were observed in the same areas as implicated here in learning, that is, in the left lateral pre- and postcentral gyrus and superior temporal gyrus (45). These same areas are also active during unperturbed speech. In a direct comparison of shifted and unshifted speech, similar activity was observed both in the primary auditory cortex and STG, as well as the precentral and postcentral gyrus, supplementary motor area, the parietal operculum, and the right inferior frontal gyrus (44). Of these areas, lesions to STG and S1 are known to lead to speech impairment, suggesting their causal involvement in the process (46). The present study establishes that these same areas are also causally involved in speech motor learning and retention, implicating their involvement in error processing and memory storage.

Delayed auditory feedback is another experimental model to assess error processing in speech. When delayed auditory signals were applied to patients undergoing direct cortical recording, changes in activity were observed in the pre- and postcentral gyrus, STG, supramarginal gyrus, and the inferior frontal gyrus. However, only STG, supramarginal gyrus, and dorsal precentral gyrus showed error related processing after correcting for differences in speech duration (47). Some of the activity differences between that work, and other studies of speech and speech learning may be attributable to cognitive engagement associated with the delayed auditory manipulation. Some of the same areas were implicated in the responses of patients with poststroke aphasia to altered auditory feedback. At the onset of perturbation, superior and middle temporal gyrus damage was associated with reduced responses to altered auditory feedback, whereas later in the perturbation, damage to the supramarginal and inferior frontal gyrus accounted for a reduced response (48). Specific tests for the causal involvement of these areas in cognitive aspects of speech motor learning and retention remain to be undertaken.

### Motor Cortical Changes in Motor Learning and Memory.

A key finding from the current study is that disruption of the primary motor cortex resulted in speech motor memory retention that was no different than that observed in a no TMS control condition. This finding, in particular, is consistent with previous reports showing no effect on retention following TMS over M1 in limb motor learning tasks (17, 18, 26). It is noteworthy that these findings suggest little functional involvement of M1, the primary motor hub of the brain, in the initial maintenance of motor memories of a newly learned task. These findings are in contrast with those from the rodent literature where clear evidence of plasticity and new dendritic spine formation in M1 is observed following motor learning (49, 50). One possibility in accounting for this discrepancy is a temporal separation in the involvement of various brain regions during motor learning and memory retention. That is, M1 could still be involved in the maintenance of learning but perhaps at a later point in the process, and/or with more challenging task requirements. For instance, studies showed that M1 plasticity with motor learning in rodents involved extensive training of a whole-body movement (although predominantly moving the limbs) for a prolonged duration (49, 50). Within the first 24 h, dendritic spine formation was observed following learning, but the newly formed spines were also observed to undergo extensive pruning, leading to only a small fraction of new spines surviving in the process (49, 50). Consequently, had it been a more demanding task with prolonged duration of learning sessions, one may also observe impairment in retention of the products of motor learning following TMS over M1. Moreover, findings from a previous study, which show changes in S1 preceding those in M1 during a motor learning task in humans are consistent with the notion of temporally separable involvement of sensory and motor cortical activity (51). It is important to note that the lack of M1 involvement in motor memory immediately following learning affords us the ability to probe and characterize the contribution of other cortical nodes (such as STG and S1) to motor learning and retention, and to rule out the possibility that the impairment observed is due to the indirect activation of M1 since direct stimulation of M1 has no effect. In this regard, the no TMS control condition in this study provides a clean probe of motor memory retention in absence of any TMS.

Evidence suggesting a role of M1 in speech motor learning has been presented previously (4, 5, 52). However, when M1 is disrupted prior to learning, as in these studies, the disruption may have affected speech performance itself and produced an impairment in learning and memory as a consequence. Another difficulty in interpreting a stimulation effect to M1 on speech motor learning is when S1 stimulation is absent. In this case, an impairment observed in speech motor learning and/or retention following M1 disruption could be confounded by the indirect disruption to neighboring S1 via leakage current and reciprocal connections (19, 36, 53–60). The disruption to M1 following speech motor learning, as in the current experimental design, ensures that only the products of learning are affected without influencing the ability to produce the primary speech motor behavior. Similarly, inclusion of S1 disruption further addresses the possibility of leakage current confounding the results and provides a cleaner assessment of neural substrates underlying speech motor learning and memory. Lastly, when evaluating motor memory, the retention test was done 24 h after learning to avoid the possibility that any impairment observed might have been transient. An earlier study (61) involving learned hand movements found that disruption of M1 immediately following learning led to an impairment in retention. However, in subsequent reports (62, 63), when retention was tested 24 h after learning, there was no evidence of an ongoing impairment. Consequently, a 24-h delay was considered appropriate to evaluate motor memory stabilization. Moreover, previous work showed similar speech motor memory retention 8- and 24-h following learning, suggesting that evaluating motor memory retention even 8 h later might have led to similar findings (12).

Although the present study found no evidence of M1 participation in speech motor memory retention, premotor areas, which were not tested here, may fulfill this role. Neuroimaging evidence suggests learning-related changes in premotor areas and rostral parietal cortex (as was observed here) in both speech and motor sequence learning (1–3, 64). These two areas are also active in preparation for movement (65), suggesting that the learning related changes could also contribute functionally to behavior.

Another noteworthy finding from this study is the sustained impairment in speech motor performance during relearning on the second visit. Although the relearning performance converges to a statistically similar extent toward the end of the second day, it can be seen that the impairment in relearning is more or less sustained for the STG and S1 conditions throughout. There are two possibilities which might account for this: First, TMS could have impaired the information passed from day-1 to day-2 which, in turn, placed a limit on relearning (66). This could include processes which subserve both error-corrective and persistent memory mechanisms. An error corrective mechanism has been shown to contribute to savings—a phenomenon which enables faster relearning of a previously learned task when compared to de novo learning of that task (66). On the other hand, a persistent component of memory is more robust to the passage of time and is reflected in performance at the beginning of the retention test (66). It is possible that TMS-based disruption affecting both—an error corrective and persistent memory mechanism could underlie the impairment in relearning and long-term retention of speech motor memory observed on the second visit.

A second possibility is that the persistent impairment might be a consequence of long-lasting effects of multiple rounds of cTBS, as were used here. Nothing comparable has been reported elsewhere. However, evidence from the clinical literature indicates that continuous theta-burst stimulation can induce effects that persist well beyond the stimulation session when applied across days. Sustained behavioral or clinical effects have most commonly been reported following multisession cTBS protocols (67–75). Although these clinical protocols involve substantially longer treatment courses and higher cumulative doses than the present study, they highlight that repetitive exposure to theta-burst stimulation contributes to longer-lasting effects. The present experiment employed two temporally spaced rounds of cTBS within a single visit, each consisting of 600 pulses delivered in theta-burst format, thereby constituting a brief form of repetitive stimulation. The sustained impairment in relearning, observed on the second visit, is consistent with the possibility that cTBS includes longer-term effects on cortical function in the auditory and somatosensory areas.

### Effects of TMS on the Unperturbed Formant and on Speech Production Itself.

Because most of the acoustical energy during vowel production is contained within the first two formant frequencies (F1, F2), their covariation has a nearly bidirectional, one-to-one correspondence with the vowel being spoken. Despite the F1-F2 covariation, it has been proposed that the control of the two formants may be independent and separable (76–78). Further, it has been observed that the F2 changes can co-occur with F1 during learning but without influencing the retention of learned F1 (12). In the present study, we considered two potential sources of influence on F2: the F1 perturbation, and TMS-based disruption. We observed little change in the F2 which was neither systematic across TMS conditions nor across the two visits. This indicates minimal involvement of F2 in this study.

Lastly, we observed no consistent or systematic changes in speech parameters (formants F1 and F2) during speech-masked noise feedback trials during baseline and immediately prior to assessing retention. This suggests that there is little effect of TMS on speech production per se. This is consistent with previous findings in the absence of TMS that showed similar performance in noise feedback trials during baseline and immediately prior to retention (12).

## Conclusion

Taken together, the findings establish that speech motor memory is impaired when sensory cortex is disrupted and is not dependent on changes in the primary motor cortex. Speech motor learning and memory is thus substantially sensory in nature.

## Data Availability

Data shown in Figs. 2–4 on which the statistical analyses were conducted have been deposited in OSF (79). Since speech acoustical data cannot be anonymized, only processed data are available.
